# Chitinase 3-like-1 is produced by human Th17 cells and correlates with the level of inflammation in juvenile idiopathic arthritis patients

**DOI:** 10.1186/s12948-016-0053-0

**Published:** 2016-11-08

**Authors:** Manuela Capone, Laura Maggi, Veronica Santarlasci, Maria Caterina Rossi, Alessio Mazzoni, Gianni Montaini, Rolando Cimaz, Matteo Ramazzotti, Marie Pierre Piccinni, Giusi Barra, Raffaele De Palma, Francesco Liotta, Enrico Maggi, Sergio Romagnani, Francesco Annunziato, Lorenzo Cosmi

**Affiliations:** 1 Department of Experimental and Clinical Medicine and DENOTHE Center, University of Florence, 50134 Florence, Italy; 2 Anna Meyer Children’s Hospital and University of Florence, 50134, Florence, Italy; 3 Dept. of Biomedical Experimental and Clinical Sciences “Mario Serio”, University of Florence, 50134 Florence, Italy; 4 Dept. of Clinical & Experimental Medicine, Second University of Naples, 80131 Naples, Italy; 5 Institute of Protein Biochemistry, CNR, 80131, Naples, Italy; 6 Department of Internal Medicine, University of Florence, Viale Pieraccini 6, 50134 Florence, Italy

**Keywords:** CHI3L1, Th17 cells, CD161, SF, JIA, CRP

## Abstract

**Background:**

CHI3L1 is a chitinase-like protein without enzymatic activity, produced by activated macrophages, chondrocytes, neutrophils. Recent studies on arthritis, asthma, and inflammatory bowel diseases suggest that chitinases are important in inflammatory processes and tissue remodeling, but their production by human T cells, has never been reported.

**Methods:**

A microarray analysis of gene expression profile was performed on Th17 and classic Th1 cell clones and CHI3L1 was found among the up-regulated genes on Th17 cells. Different types of helper T cell clones (TCCs) were then evaluated by Real Time PCR (RT-PCR) for CHI3L1 mRNA expression; protein expression was investigated in cell lysates by western blotting and in cultures supernatants by ELISA. ELISA was also used to measure CHI3L1 in the serum and in the synovial fluid (SF) of juvenile idiopathic arthritis (JIA) patients.

**Results:**

At mRNA level CHI3L1 was highly expressed by Th17, Th17/Th1, non classic Th1 and even in Th17/Th2 cell clones, whereas it was virtually absent in CD161− classic Th1 and Th2 TCCs. CHI3L1 was also detected in cell culture supernatants of Th17 and Th17-derived cells but not of classic Th1. Moreover CHI3L1 was higher in the SF than in serum of JIA patients, and it positively correlated with the frequency of Th17 and non-classic Th1 cells in SF. CHI3L1 in SF also positively correlated with the C reactive protein (CRP) serum levels, and with the levels of some proinflammatory cytokines, such as IL-6 and p40, which is the common subunit of IL12 and IL23.

**Conclusions:**

Here we describe for the first time CHI3L1 production by T cells owing the Th17 family. Moreover the positive correlation found between the frequency of Th17 and Th17-derived cell subsets and CHI3L1 levels in SF of JIA patients, in agreement with the suggested role of these cells in inflammatory process, candidates CHI3L1 as a possible biological target in JIA treatment.

## Background

Chitinases belong to a family of hydrolases, the glycosyl hydrolase 18 proteins (GH18), characterized by the ability to cleave the environmental polysaccharide chitin, present in the coating of many pathogens including protozoan parasites, fungi, and nematodes. Although mammals do not synthesize chitin, they do synthesize chitinases and chitinase-like proteins (CLPs), that, due to mutations in their active domain, don’t have chitinolytic enzyme activity. Members of this family include chitinase 3-like-1 (also known as YKL-40 or HC-gp39) [1], chitotriosidase [2], YKL-39 [3], Ym1 [4], acidic mammalian chitinase (AMCase) [5], oviduct-specific glycoprotein [6], and stabilin-1-interacting chitinase-like protein [7]. The physiological role of these secretory proteins remains unclear and debated, even if recent findings strongly support the concept that chitinases and CLPs are induced at sites of infection as part of an innate anti-pathogen response: in that context they are described to act by controlling tissue injury and specific innate immune response such as those induced by chitin fragments and augmenting adaptive immune responses, ensuring pathogen eradication [8]. The majority of the glycosyl 18-hydrolase family member are CLPs and among them chitinase 3-like-1 (CHI3L1) is the most studied: it is endogenously expressed by several cell types including macrophages, neutrophils, chondrocytes, fibroblasts, endothelial cells and colonic, ductal, and airway epithelial cells [1, 8]. CHI3L1 expression is upregulated during inflammatory conditions when it is essential for mounting Th2-type immune responses, stimulating dendritic cell accumulation/activation and recruitment of macrophages but also for inhibiting eosinophils, macrophages and T cells death-receptor mediated, apoptosis [9]. CHI3L1 has been also shown to stimulate proliferation of human connective tissue cells through a MAPK/AKT dependent pathway [10]. In agreement with these evidences on CHI3L1 activities, KO mice have been generated that developed less severe inflammatory responses at the acute phase of bacterial infectious colitis [11] as well as suppressed Th2-type immune responses, characterized by lower tissue inflammation, fibrosis and higher immune cell apoptosis [9]. Increased levels of CHI3L1 protein and/or mRNA have been shown in patients with a broad spectrum of diseases including asthma [9, 12], inflammatory bowel disease (IBD) [13, 14] and arthritis [15, 16] where they reflect the activity and natural history of the disease. In fact, genetic variation of CHI3L1 gene associated with its higher expression in serum and with exacerbation of asthma and airways tissue remodelling [17]. Finally CHI3L1 has been proposed as a candidate autoantigen in rheumatoid arthritis (RA) [18] due to its different expression in cartilage and synovial tissues, and to a possible link with the human leukocyte antigen (HLA)-DR4 shared epitope [19]. As a result of this knowledge CHI3L1 is already considered a prognostic biomarker for several immune mediated diseases and has been proposed to be a therapeutic target in conditions characterized by fibrosis, extracellular matrix remodeling and acute or chronic inflammation [12, 14–16].

It is already well described that T helper populations, in addition to their protective role, are involved in several chronic inflammatory immune-mediated disorders. CD4+ T helper (Th) cells can be classified into lineages on the basis of the cytokines production, the specific transcription factors expression and the immune-physiological function they mediate. Th1 cells, which express the transcription factor T-box expressed in T cells (T-bet) and secrete IFN-γ, protect the host against intracellular infections; Th2 cells, which express GATA-3 and secrete IL-4, IL-5 and IL-13, mediate host defense against helminths [20, 21]. Recently, additional subsets that preferentially produce distinct cytokines have been described. The most studied subset includes cells that selectively produce IL-17A (Th17 cells), express the transcription factor RAR-related orphan receptor (ROR)γt, the IL-23 receptor (IL-23R), the chemokine receptor CCR6 and the lectin receptor CD161 and protect the host against extracellular pathogens infections [22–24]. Human Th17 cells originate from CD161+ precursors present in umbilical cord blood (UCB) and new born thymus, and maintain CD161 expression, that is detectable on memory circulating and tissue-infiltrating Th17 lymphocytes [24–26]. Th17 lymphocytes, beyond their protective role in the clearance of extracellular pathogens, also play a role in the pathogenesis of several autoimmune and inflammatory diseases [27]. In particular, a determinant role for Th17 cells has been proposed in multiple sclerosis, RA and IBDs, but also in psoriasis and contact dermatitis [28, 29], under-evaluating the contribution of Th1 cells [30], previously shown to be crucial. Although classically viewed as distinct lineages, recent evidence indicate that Th17 cells are more plastic than previously thought. It is not fully understood how often such plasticity occurs in the course of physiologic responses to pathogens and what its importance is in protective immunity, but in inflammatory conditions Th17 lymphocytes, that have shifted towards a Th1 or Th2 phenotype maintaining CD161 and RORc expression and acquiring the ability to produce IFN-γ or IL-4, seem to be particularly aggressive and more pathogenic than the unshifted ones [31, 32]. In a recent study, we found that Th17 cells are rare in the SF of patients with JIA whereas Th1 cells were highly predominant, which was at least partially due to the property of Th17 cells to shift into Th1 cells in presence of IL-12, and/or TNF-α [33, 34]. The Th17-derived Th1 cells expressed CD161, while the other Th1 cells present in the SF did not, and we named the former cells as non-classic, as compared with classic CD161− Th1 cells [33]. Moreover, we found an accumulation of Th17 and non-classic CD161+ Th1 lymphocytes in fistula curettage of patients suffering of fistulising Crohn’s disease [35, 36]. Another evidence of the high plasticity of human Th17 cells also emerges by the recent finding of the existence of a subset of human circulating memory CD4 T cells that produce both IL-17A and IL-4 [31]. This previously unknown population of Th17/Th2 lymphocytes maintains CD161 membrane expression, is more represented in the circulation of patients with allergic asthma than in healthy donors, and is enriched in cells specific for the sensitizing allergen, suggesting a possible role in the pathogenesis of the disease [31]. In the present study, we found that human Th17, and non-classic Th1 cells are able to produce CHI3L1, whereas classic Th1 cells are not. In agreement with the suggested role in inflammatory processes we also found CHI3L1 in the SF of JIA patients, which positively correlated to the inflammatory status. These data open new insights about the possible involvement of CHI3L1 in those chronic inflammatory disorders in which Th17 subsets play a pathogenic role.

## Methods

### Reagents

The culture media used was RPMI 1640 (Seromed) supplemented with 2 mM l-glutamine, 1% non-essential amino acids, 1% sodium pyruvate, 2 × 10^−5^ M 2-mercaptoethanol (2-ME; all from Invitrogen), and 10% FBS HyClone (Gibco Laboratories, Grand Island, NY). Unlabeled or fluorochrome-conjugated anti- CD3, CD4, CD8, CD161, CCR6, IFN-γ, and isotype-matched control mAb were purchased from BDBiosciences (San Jose, CA, USA). The fluorochrome-conjugated anti-IL-17 mAb was obtained from eBioscience (San Diego, CA, USA) and the fluorochrome-conjugated anti-CXCR3 mAb was obtained from R&D Systems (Minneapolis, MN, USA). PMA, ionomycin and brefeldin A were purchased from Sigma Chemical Co. (St. Louis, MO, USA). Activating mAb anti-CD3 and -CD28 were purchased from BDBiosciences (San Jose, CA, USA).

### Establishment of T cell clones and their characterization

CD4+ T cells, derived from PBMNC of healthy donors by using the CD4 isolation kit II (Miltenyi Biotec, Bergisch Gladbach), were further divided into CD161+ and CD161− T-cell fractions by a staining with an anti-CD161–PE mAb, followed by incubation with an anti-PE microbead mAb (Miltenyi Biotec). Then the two cell subsets CD4+CD161+ and CD4+CD161− were cultured under limiting dilution (0.3 cell/well) in presence of 10^5^ irradiated (9000 rad) allogeneic PBMCs as feeder cells, 1% PHA (vol/vol), and 50 U/ml rIL-2 (Proleukin, Prometheus, Inc. San Diego, USA), in order to obtain T cell clones. Recovered CD4+ T cell clones were classified on the basis of their ability to produce IFN-γ and/or IL-17 and to express surface marker CD161, as previously described [24]. Briefly, T cells were polyclonally stimulated with PMA plus ionomycin, fixed in formaldehyde and then analyzed for intracellular cytokines production on a BDLSR II flow cytometry (BDBiosciences). Selected T-cell clones of each phenotype were further analysed by flow cytometry for surface expression of CXCR3A and CCR6. Seven Th17, non-classic -Th1 and classic Th1 clones were stimulated at a culture concentration of 10^6^ cells/ml for 72 h in presence of medium or mAbs anti- anti-CD3-CD28 (5 μg/ml each), then cultures supernatants were collected and stored at −30 °C for further analysis.

### Microarray

Gene expression profiles on human Th17, classic and non-classic Th1 clones were assessed by cDNA microarray technique using the Human Genome Survey Microarray (Applied Biosystems). In brief, RNA from different samples was amplified and labeled with Digoxigenin-UTP (DIG-UTP; Applied Biosystems). 10 μg of DIG-labeled cRNA was hybridized to the Human Genome Survey Microarray and read using the 1700 Chemiluminescent Microarray Analyzer (Applied Biosystems). Expression Array System software (Applied Biosystems) was used to analyze the microarrays images. Only microarrays showing a normalized signal intensity >5000 and a median background <600 were analyzed and normalized using Spotfire and Intergomics software (Spotfi re Inc.). Class comparisons expressed as Benjamini–Hochberg false discovery rates were done using parametric tests (LIMMA) after log transformation. Each sample was analyzed three times. Microarray data are available from the Gene Expression Omnibus database (http://www.ncbi.nlm.nih.gov/gds) under the accession number GSE30664.

### Real-time quantitative RT-PCR

Taq-Man RT-PCR was performed, as described elsewhere [24]. Quantitative PCR analysis of CHI3L1 was performed by using primers and probes purchased from Applied Biosystems (Warrington, UK). Quantification was performed on cell number.

### Western Blot analysis

T Cell lysates were prepared in lysis buffer (RIPA buffer: 25 mM Tris–HCl pH 7.6, 150 mM NaCl, 1% NP-40, 1% sodium deoxycholate, 0.1% SDS and 5 mM EDTA) containing a protease- and phosphatase-inhibitor mixture (Pierce). Proteins were quantified by Bradford Assay before equivalent amounts were separated by SDS-PAGE were run on a 4–20% polyacrylamide gel (Bio-Rad, USA) for 2 h at 120 V, transferred to a nitrocellulose membrane for 16 h at 30 V, blocked in 5% milk for 1 h at room temperature. CHI3L1 protein expression was detected by anti-human CHI3L1 rabbit-pAb (ab88847; Abcam) administered for 18 h followed by incubation with horseradish peroxidase–conjugated anti-rabbit pAb (Santa-Cruz biotechnology) for 1 h at room temperature. An enhanced chemiluminescent substrate (GE Healthcare) for detection of HRP was used for visualization.

### Subjects

Samples of PB and SF were obtained from 25 children, 15 average aged, with oligoarticular-onset JIA, whose diagnosis was made in accordance with the International League of Associations for Rheumatology classification criteria for JIA [37]. Patients with systemic-onset disease, polyarticular JIA, or enthesitis-related arthritis were excluded, in order to assess a homogeneous patient group. The C-reactive protein (CRP) serum levels (normal values up to 0.5 mg/dl), assessed using the particle-enhanced turbidimetric immunoassay method, were evaluated by staff in the service laboratory of the Anna Meyer Pediatric Hospital in Florence, Italy.

The procedures followed in the study were in accordance with the ethics standards of the Regional Committee on Human Experimentation. Informed consent was obtained by parents or guardians.

### Quantitative detection of CHI3L1

CHI3L1 levels in the culture supernatants, sera and SF of JIA patients were assessed using a commercially available ELISA kit by R&D Systems. Assays were performed following manufacturer’s instructions.

### Enzyme-linked immunosorbent assays (ELISAs)

All the cytokines analyzed in the sera and SF of JIA patients were assessed using a Custom LEGENDplex ELISA kit from BioLegend whereas the detection of IL12p40 levels was done by an ELISA kit from Invitrogen.

### Statistical analysis

A non-parametric Mann–Whitney test, performed by Origin Pro 2015 software, was used for statistical analysis. p values less than or equal to 0.05 were considered significant. Pearson’s correlation coefficients were used to calculate the correlations.

## Results

### CHI3L1 is produced by non-classic Th1 and Th17 clones

**Fig. 1:**
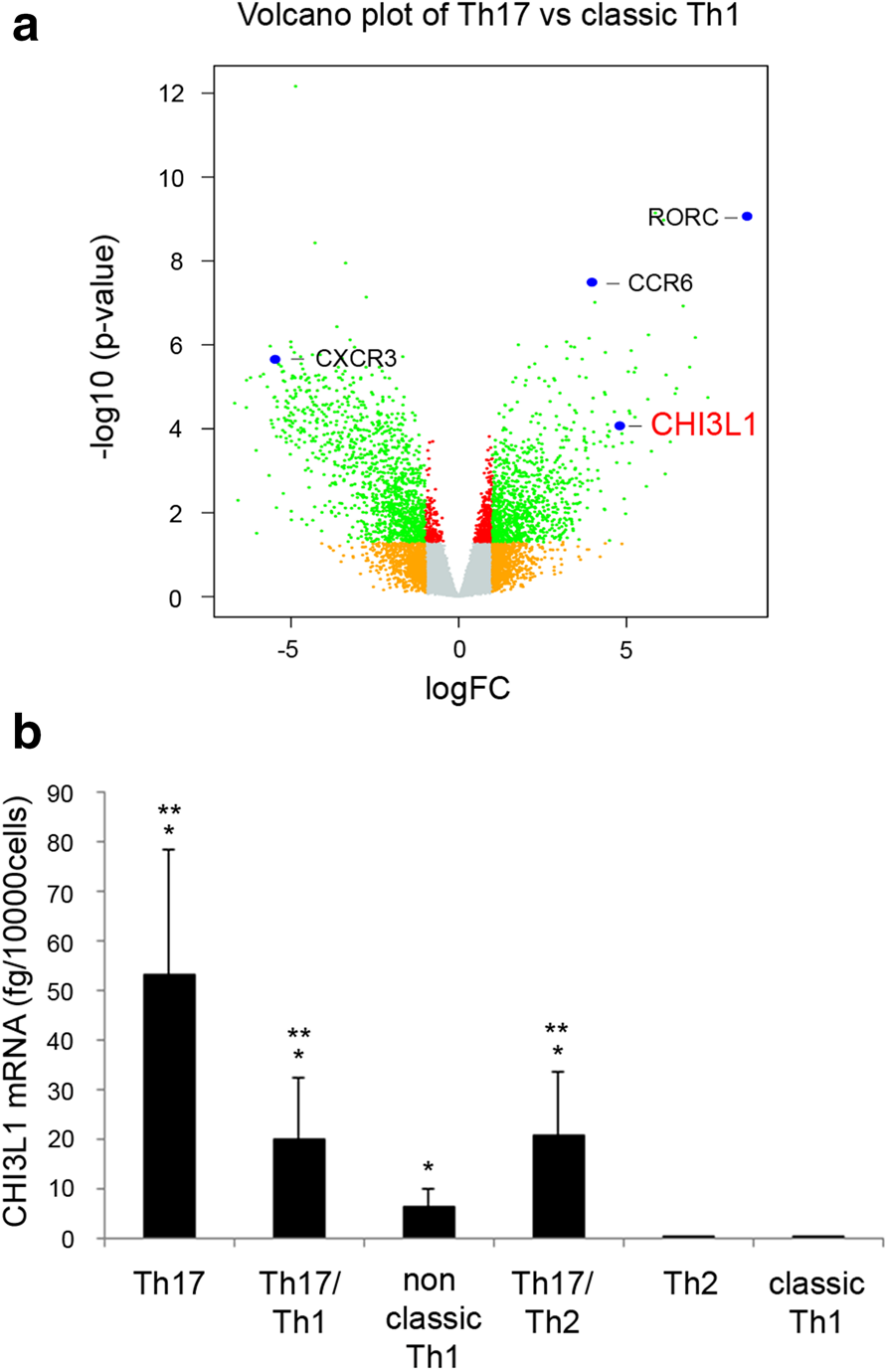
CHI3L1 is expressed by Th17 and Th17-derived cells. **a** Volcano plot representation (log fold change vs. −log 10 p value) of Th17 and classic Th1 cells gene expression profiles. **b** CHI3L1 mRNA expression was evaluated by real time quantitative RT-PCR on Th17 (n = 14), Th17/Th1 (n = 7), non classic Th1 (n = 9), Th17/Th2 (n = 8), Th2 (n = 5) and classic Th1 (n = 15) clones. *Columns* represent mean values (±SE). *p ≤ 0.05 compared to classic Th1 column; **p ≤ 0.05 compared to Th2 column

In previous studies, we generated a series of classic Th1 and Th17 clones from circulating CD4+ T cells obtained from healthy donors. In order to identify the differences in the gene expression profile between Th17 and classic Th1 clones, a microarray analysis was performed [24, 26, 38, 39]. Among genes that fulfilled the criteria described in the Methods section as up- or down-regulated genes in Th17 versus classic Th1 clones, in addition to those expected on the basis of previous knowledge, such as*, RORγt*, *CCR6, CXCR3, CD161*, *IL*-*17, IL*-*23R and IL4I1* [40, 41], there was also *CHI3L1* (Fig. 1a). Therefore, CHI3L1 expression was analysed by quantitative-RT PCR and extended to a new panel of human Th17, Th17/Th1, non-classic Th1, Th17/Th2 and Th2 clones, derived from healthy subjects. As shown in Fig. 1b, Th17, Th17/Th1, non-classic Th1 and Th17/Th2 clones expressed significantly higher levels of CHI3L1 mRNA than classic Th1 and Th2 clones.

**Fig. 2:**
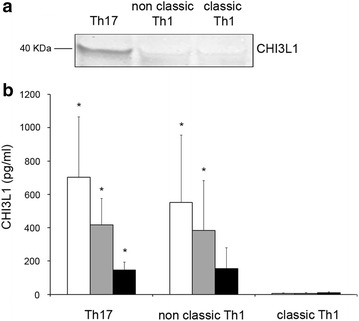
CHI3L1 protein is secreted by Th17 and non-classic Th1 cells. **a** CHI3L1 protein (40 kDa) expression was evaluated on cell lysates of Th17, non classic-Th1 and classic-Th1 clones by western blot analysis. **b** Secreted CHI3L1 was measured on cell culture supernatants of Th17 (n = 7), non classic Th1 (n = 7) and classic Th1 (n = 7) clones after 72 h stimulation with medium (*white columns*), anti-CD3/CD28 mAbs (*grey columns*) or PMA plus Ionomycin (black columns) by ELISA. Columns represent mean values (±SE). *p ≤ 0.05

Based on the results obtained at mRNA level, we first assessed CHI3L1 expression at protein level by western blot analysis on resting TCCs lysates, confirming that Th17 and non-classic Th1 clones express CHI3L1 while classic Th1 did not (Fig. 2a). Then, in order to understand if Th17 and Th17-derived T cells, like non-classic Th1 clones were also able to secrete CHI3L1, TCCs of each phenotype were cultured in vitro for 72 h in the absence or presence of anti-CD3 plus anti-CD28 mAbs or PMA plus ionomycin and the cell culture supernatants collected. As shown in Fig. 2b, Th17 and non-classic Th1 clones produced comparable amounts of CHI3L1 independently of anti-CD3 plus anti-CD28 mAbs or PMA ionomycin stimulation, whereas, accordingly to mRNA data expression, classic Th1 cells did not produce CHI3L1. Interestingly we observed a reduction in CHI3L1 production by both Th17 and non-classic Th1 cells in the stimulating conditions (Fig. 2b).

### The amount of CHI3L1 present in the SF of JIA patients positively correlates with the frequency of Th17 cells

**Fig. 3:**
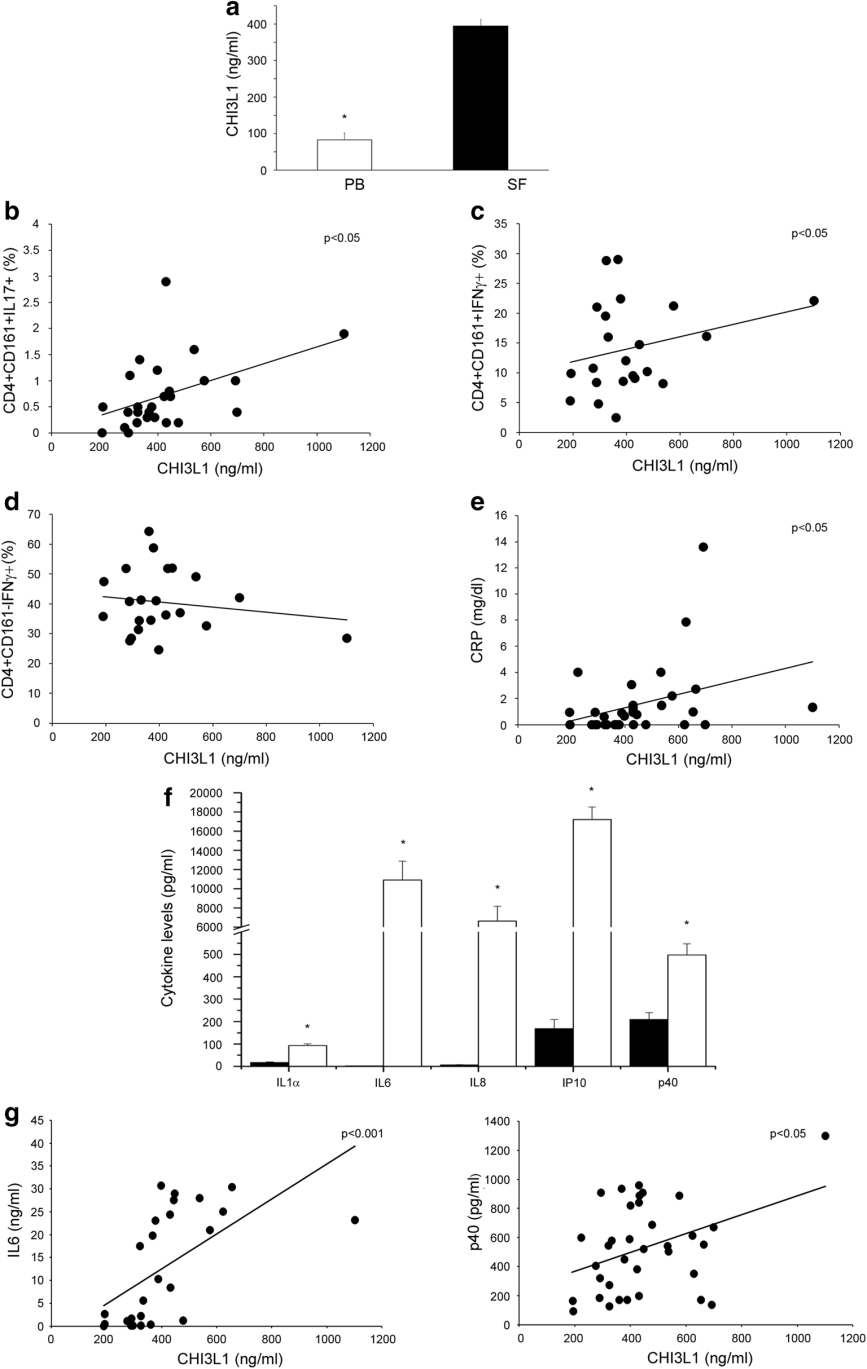
CHI3L1 levels positively correlate with the frequency of CD161+ cells in the SF of JIA. PB and SF from 25 JIA patients were evaluated: sera from peripheral blood (*white columns*) and SF (*black columns*) were obtained and CHI3L1 amounts evaluated by ELISA (**a**). *Columns* represent mean values (±SE). *p ≤ 0.05. MNC cells obtained from SF were characterized by flow cytometry and correlation between CHI3L1 levels in SF and frequency of CD4+CD161+IL17+ cells (**b**), CD4+CD161+IFNγ+ cells (**c**) and CD4+CD161−IFNγ+ cells (**d**), in SF, was represented. **e** Correlation between CHI3L1 levels in SF and CRP serum levels in the same group of patients. **f** The indicated cytokines were assessed in the sera (*white columns*) and SF (*black columns*) from 25 JIA patients. *Columns* represent the mean values (±SE). *p ≤ 0.05. **g** Correlation between CHI3L1 levels in SF and IL6 (*left panel*) or p40 (*right panel*) SF levels in the same group of patients, was represented

It has been shown that, in RA patients serum, CHI3L1 levels positively correlated with levels of C reactive protein (CRP) [42, 43]. We have recently shown that the frequency of CD4+CD161+, found among CD4+ T cells present in the SF of JIA patients, positively correlates with the levels of two inflammatory parameters, such as the erythrocytes sedimentation rate (ESR) and the CRP, measured in the sera of the same patients [33]. In order to understand if CHI3L1 produced by Th17 cells may play a role in JIA pathogenesis, we evaluated both the CHI3L1 levels and the frequencies of the different T helper cell populations in the synovial fluid of a cohort of 25 JIA patients. As shown in Fig. 3a, the CHI3L1 amounts present in the SF were significantly higher than those observed in the sera of the same patients. Moreover, and more importantly, the CHI3L1 amounts present in the SF of JIA patients positively correlated with the frequencies of Th17 (Fig. 3b) and non-classic Th1 (Fig. 3c) cells, but not with the classic Th1 (Fig. 3d) cells. Finally CHI3L1 quantity in the SF of JIA patients positively correlated with the CRP levels present in the sera of the same patients (Fig. 3e). To further strengthen the association between CHI3L1 levels and the inflammatory status, we checked if these levels correlate also with inflammatory cytokines, in SF. To this end we firstly compared the levels of inflammatory cytokines in peripheral blood and SF of JIA patients (Fig. 3f). Among the inflammatory cytokines that resulted to be more expressed in SF than PB, we found a positive correlation of CHI3L1 levels with both IL6 and the p40 chain (Fig. 3g).

## Discussion

In this paper we detected for the first time the expression of CHI3L1 in human T cells. As a result of a gene expression profile microarray analysis on T cell clones we unexpectedly found that CHI3L1 was one of the most up-regulated genes, together with other described (i.e. CCR6 and RORc), in resting Th17 clones when compared to classic Th1. We and others already assessed that the frequencies of CD161+ Th17-derived Th1, Th17/Th1 and Th17/Th2, cells, positively correlated with severe phenotype of different chronic inflammatory disorders such as JIA, IBDs and asthma, respectively [31–36]. In the same pathological conditions higher levels of CHI3L1 have been detected in patients’ serum respect to healthy donors [14–16]. Based on this knowledge CHI3L1 mRNA expression was evaluated confirming the microarray data. We found that CHI3L1 mRNA was selectively expressed by human Th17, Th17/Th1, non-classic Th1, and Th17/Th2, cells but not by their CD161− counterparts. This finding means that CHI3L1 is expressed only by CD161+ cells. Of note, mRNA amounts of CHI3L1 were highest on pure Th17 cells and showed a trend to decrease in the other CD161+ subsets, suggesting that it could represent a sort of marker of Th17 lymphocytes. The finding that Th17/Th2 cells express high levels of CHI3L1 is in agreement with the possible pathogenic role of these cells in neutrophilic steroid-resistant asthma since both Th17/Th2 and serum levels of CHI3L1 result to be increased in such disease [42–44].

In the second part of the study, we focused on the three cell subsets Th17, non-classic Th1, as the main example of Th17-derived phenotype, and classic Th1 cells, performing western blot analysis of CHI3L1 protein expression on resting T cell lysates. Results showed an evident expression of CHI3L1 on Th17 cells even if on non-classic and classic Th1 it seemed virtually absent. This was apparently in contrast with the data obtained at mRNA level and in order to explain this discrepancy we moved to investigate CHI3L1 level cultures supernatants. CHI3L1 levels were measured by ELISA on supernatants obtained by culturing Th17, non-classic Th1 and classic Th1 clones in presence or absence of two types of polyclonal stimulus (mAbs anti-CD3/CD28 or PMA plus ionomycin). As expected CHI3L1 was produced only by Th17 and non-classic Th1 cell cultures whereas in classic Th1 ones it was not detected. This discrepancy between western blot analysis and ELISA, could be due to the fact that CHI3L1 is a secreted protein. Probably in Th17 cells it is anyway detectable, because it is stored in the intracellular compartment, whereas the same did not occur in non-classic Th1 cells where the amounts are lower. Recent studies demonstrated that increased levels of CHI3L1 in synovial fluid and serum have been found in patients with active RA compared to patients with inactive RA and healthy subjects, and changes in serum CHI3L1 during drug therapy reflect changes in disease activity. Moreover, in patients with early RA, a continuously elevated serum CHI3L1 is associated with progression of joint destruction [15, 16]. Given this literature we deepened our results on TCCs and considering our previous evidences on the involvement of Th17 and Th17-derived Th1 cells in JIA pathogenesis [33] we searched for any possible correlation between them and CHI3L1 expression. To this end we first assessed CHI3L1 levels on sera and synovial fluids from several JIA patients and showed that they are significantly higher at the inflamed tissue when compared to the periphery, thus confirming literature data on rheumatoid arthritis [45, 46]. Moreover we characterized SF for the presence of CD161-expressing cells and found that the frequencies of CD161+IL17− or IFNγ− producing cells positively correlated to CHI3L1 levels. This led us to hypothesize that CHI3L1 production by Th17 and non-classic-Th1 cells may be somehow related to their pathogenic role in JIA. Accordingly, in the same patients CHI3L1 levels in the synovial fluid were also positively correlated with the inflammatory status evaluated by CRP. Moreover, also the levels of proinflammatory cytokines, such as IL6 and the p40 chain, positively correlated with CHI3L1 in SF. The p40 chain is the common subunit of IL12 and IL23, whose role in the induction of the Th1 (both classic and non-classic) and Th17 effectors has been clearly shown [24]. In this view the finding of p40 overexpression in SF was not surprising, and in agreement with the proposed role of these T cell subset in JIA pathogenesis.

Our data are in agreement with the most accepted concept of CHI3L1 as a marker of disease activity and inflammation in several immune-mediated disorders. The previously unknown information that CHI3L1 is produced by Th17 and Th17-derived cells, open new insights on the possible role of this molecule in those inflammatory diseases in which these T cell subsets play a pathogenic role.

## Conclusion

CHI3L1 is produced by Th17 and non-classic Th1 cells, already demonstrated to exert a role in the pathogenesis of JIA. CHI3L1 levels on SF from JIA patients positively correlated with frequencies of Th17-derived cells, with CRP levels and with the levels of IL6 and the p40 IL12/IL23 subunit in SF. These findings suggest a possible involvement of CHI3L1 in mediating joint inflammation in JIA. Based on these findings CHI3L1 could be considered a possible biological target for medical treatment of JIA.

## Abbreviations

CHI3L1: chitinase 3-like-1
CLPs: chitinase-like proteins
IBD: inflammatory bowel diseases
RA: rheumatoid arthritis
JIA: juvenile idiopathic arthritis
SF: synovial fluid
CRP: reactive protein C
